# Adaptive weighted progressive iterative approximation based on coordinate decomposition

**DOI:** 10.1371/journal.pone.0317225

**Published:** 2025-01-29

**Authors:** Yushi Liu, Yan Wang, Chengzhi Liu

**Affiliations:** 1 School of Mathematics and Finance, Hunan University of Humanities, Science and Technology, Loudi, China; 2 School of Information, Hunan University of Humanities, Science and Technology, Loudi, China; Federation University Australia, AUSTRALIA

## Abstract

During the iterative process of the progressive iterative approximation, it is necessary to calculate the difference between the current interpolation curve and the corresponding data points, known as the adjustment vector. To achieve more precise adjustments of control points, this paper decomposes the adjustment vector into its coordinate components and introduces a weight for each component. By dynamically adjusting these weights, we can accelerate the convergence of iterations and enhance approximation accuracy. During the iteration, the weight coefficients are flexibly adjusted based on the error of the current iteration step, demonstrating the flexibility and precision of the geometric iterative method in addressing geometric approximation problems. Numerical experiment results indicate that this vector decomposition technique is a critical mathematical operation for improving algorithm efficiency and precisely adjusting the shapes of curves or surfaces to approximate the data set.

## 1 Introduction

Progressive iterative approximation (PIA) is an interpolation-based geometric iterative method. The basic idea of PIA is to continuously adjust the control points of curves and surfaces so that the resulting limit curves and surfaces interpolate the given set of data points [1, 2]. In the iteration of PIA, new control points are calculated based on the current control points and the data points to be interpolated. This process is repeated until the generated curves or surfaces meet specific accuracy requirements or reach a predetermined number of iterations. This method has clear geometric significance and is easy to understand and implement. More importantly, since it adopts an iterative approach, it avoids constructing complex linear systems, thereby reducing the complexity of the solution. In certain scenarios requiring high-precision fitting, interpolation-based geometric iterative methods have significant advantages and wide applications, see [2].

In recent years, researchers have made various improvements to the PIA method for data interpolation to enhance its computational efficiency and accuracy. For example, in [3], the introduction of weights led to the weighted PIA method, known as WPIA; the PIA with multiple weights [4, 5] uses different weights for different adjustment vectors, allowing more flexible handling of local detail features of curves/surfaces and enhance the accuracy; the Chebyshev polynomial accelerated iterative method [6], the preconditioned PIA method [7, 8], and the PIA method with shape parameter curves [9], the iterative construction methods based on the splitting of the collocation matrix, including Jacobi–PIA [10, 11], GS–PIA [12–14], and SOR–PIA [15], as well as other methods [16], etc.

In the aforementioned research findings on the acceleration of geometric iterative methods, some methods are only applicable to specific basis functions. For instance, the method presented in [16] is not ideal in terms of accuracy when approximating large-scale data with Bézier curves; the methods in [7, 8] can only accelerate the PIA for basis functions such as Bernstein and Said-Ball; the methods in [10–15] can only accelerate the PIA method for B-spline curves, etc. It is worth mentioning that research on accelerating convergence by introducing weights is of great significance in geometric iterative methods. Some weighted methods, such as the WPIA method [3] and the PIA with multiple weights [4, 5], are applicable to most PIA methods for curves and surfaces with normalized totally positive basis functions, exhibiting universality. By introducing weight coefficients, the convergence and computational efficiency of the PIA method is improved, as demonstrated in [3–5], and a lot literature therein. Reasonable weights not only increase the algorithm’s convergence rate but also provide more effective tools for handling large-scale data and complex shapes. Despite the significant achievements of weighted PIA methods, there are still some issues that merit further research. For example, how to automatically select and adjust weights to meet different approximation needs, and how to extend this method to more complex geometric shapes. The selection and adjustment strategies for weight coefficients remain key focuses and challenges for future research.

In order to achieve the desired interpolation effect, the control points of the interpolation curve/surface must be continuously adjusted during the iterative process of PIA. This process is not something that can be accomplished in one step, but rather requires consideration of the components of the control vertices along all coordinate axes. However, in-depth research and numerical examples have shown that not all coordinate components need to be continuously adjusted during the adjustment process. In fact, some coordinate components remain relatively stable after several iterations and do not require further adjustment. This finding is of great significance for improving the efficiency and accuracy of iterative methods, as it allows for a greater focus on those coordinate components that truly require optimization, thereby accelerating the entire interpolation process and enhancing the quality of the results.

To rapidly adjust the control points of the interpolating curve, this paper will delve into the adjustment vector weight coefficients in the PIA method. We will adopt a strategy of coordinate decomposition and introduce adjustable weight coefficients, aiming to enhance the flexibility and precision of the PIA method. The organization of this paper is as follows: Section 2 provides a brief overview of the PIA method and its related conclusions; Section 3 presents the adaptive weighted progressive iterative approximation based on coordinate decomposition, applicable to both curve and surface cases; Section 4 demonstrates the effectiveness of the proposed method through numerical experiments. Some conclusions are drawn in the last section.

## 2 Preliminary

### 2.1 Progressive iterative approximation

Let ${\left\{p_{i}\right\}}_{i=0}^{n}$ a set of data points in $R^{2}$ or $R^{3}$ to be interpolated, with the *i*-th point is associated with a parameter *t*_*i*_. Let ${\left\{b_{i}\left(t\right)\right\}}_{i=0}^{n}$ be a blending basis that satisfies *b*_*i*_(*t*) ≥ 0 and $\sum_{i=0}^{n}b_{i}\left(t\right)=1$ for *t* ∈ [0, 1].

Initially, we use the interpolation points ***p***_*i*_(*i* = 0 ⋯ *n*) as the initial control points, that is, $p_{i}^{\left(0\right)}=p_{i}$. This allows us to derive an initial interpolation curve ${\left\{p_{i}\right\}}_{i=0}^{n}$ given by

$$\gamma^{\left(0\right)}\left(t\right)=\sum_{i=0}^{n}p_{i}^{\left(0\right)}b_{i}\left(t\right).$$

We then calculate the difference vector between the current curve and the interpolation points, $\delta_{i}^{\left(0\right)}=p_{i}-\gamma^{\left(0\right)}\left(t_{i}\right)$ for *i* = 0, 1, ⋯, *n*, and use $\delta_{i}^{\left(0\right)}$ as the adjustment vector to adjust the *i*-th control point, yielding $p_{i}^{\left(1\right)}=p_{i}^{\left(0\right)}+\delta_{i}$. Subsequently, using the new set of control points ${\left\{p_{i}^{\left(1\right)}\right\}}_{i=0}^{n}$, a new interpolation curve ***γ***^(1)^(*t*) can be generated.

Let the interpolation curve generated after *k* iterations be denoted as ***γ***^(*k*)^(*t*). The adjustment vectors for the control points can be calculated by

$$\begin{array}{c} \delta_{i}^{\left(k\right)}=p_{i}-\gamma^{\left(k\right)}\left(t_{i}\right). \end{array}$$ (1)

The interpolation curve generated in the (*k* + 1)-th iteration is given by

$$\gamma^{\left(k+1\right)}\left(t\right)=\sum_{i=0}^{n}p_{i}^{\left(k+1\right)}b_{i}\left(t\right),\;k=0,1,2,\cdots ,$$

where the updated control points are defined as

$$\begin{array}{c} p_{i}^{\left(k+1\right)}=p_{i}^{\left(k\right)}+\delta_{i}^{\left(k\right)}. \end{array}$$ (2)

Substituting $\delta_{i}^{\left(k\right)}$ from Eq (1) into Eq (2), we get

$$\begin{array}{c} p_{i}^{\left(k+1\right)}=p_{i}^{\left(k\right)}+(p_{i}-\gamma^{\left(k\right)}\left(t_{i}\right)). \end{array}$$ (3)

Thus, a sequence of sets of control points ${\left\{p_{i}^{\left(k\right)}\right\}}_{i=0}^{n}$ and a corresponding sequence of curves ***γ***^(*k*)^(*t*), for *k* = 0, 1, ⋯ can be generated. If the limit $\underset{k\to \infty }{\text{lim}}\;\gamma^{\left(k\right)}\left(t_{i}\right)=p_{i}$ holds true for each *i*, then the sequence of curves ***γ***^(*k*)^(*t*) is said to have the PIA property, and the method of generating this sequence of curves is referred to as the PIA method. As stated in [1], all normalized totally positive basis functions possess the PIA property.

Let $P^{\left(k\right)}={\left[p_{0}^{\left(k\right)},p_{1}^{\left(k\right)},\cdots ,p_{n}^{\left(k\right)}\right]}^{T}$, ***P*** = [***p***_0_, ***p***_1_, ⋯, ***p***_*n*_]^T^, and $\Delta^{\left(k\right)}={\left[\delta_{0}^{\left(k\right)},\delta_{1}^{\left(k\right)},\cdots ,\delta_{n}^{\left(k\right)}\right]}^{T}=P-BP^{\left(k\right)}$, then the process of updating control points in Eq (3) can be written in matrix form as

$$\begin{array}{c} P^{\left(k+1\right)}=P^{\left(k\right)}+\Delta^{\left(k\right)}=\left(I-B\right)P^{\left(k\right)}+P, \end{array}$$ (4)

where *I* is the identity matrix, $B={\left[b_{i}\left(t_{j}\right)\right]}_{i,j=0}^{n,n}$ is the collocation matrix of the basis functions ${\left\{b_{i}\left(t\right)\right\}}_{i=0}^{n}$ evaluated at *t*_*j*_ for *j* = 0, 1, ⋯, *n*.

### 2.2 Weighted progressive iterative approximation

To achieve faster convergence, Lu proposed the weighted progressive iterative approximation (WPIA) method [3]. This method is implemented by multiplying the difference vector $\Delta_{i}^{\left(k\right)}$ by a weight *ω* where 0 < *ω* < 2, i.e.,

$$\begin{array}{c} P^{\left(k+1\right)}=\left(I-\omega B\right)P^{\left(k\right)}+\omega P. \end{array}$$ (5)

**Lemma 1** [3] *When*

$$\begin{array}{c} \omega =\frac{2}{1+\lambda_{n}}, \end{array}$$ (6)

*the WPIA achieves the fastest convergence. In such case, the spectral radius of WPIA is*

$$\rho \left(I-\omega B\right)=\frac{1-\lambda_{n}}{1+\lambda_{n}},$$

*where* λ_*n*_ *is the smallest eigenvalue of the collocation matrix B*.

## 3 Adaptive PIA based on coordinate decomposition

### 3.1 The curve case

If ${\left\{p_{i}\right\}}_{i=0}^{n}$ is a set of points in the two-dimensional space $R^{2}$, the control point adjustment vector in Eq (3) can be decomposed along the *x*-coordinate and *y*-coordinate directions. If ${\left\{p_{i}\right\}}_{i=0}^{n}$ is a set of points in the three-dimensional space $R^{3}$, the control point adjustment vector in Eq (3) can be decomposed along the *x*-coordinate, *y*-coordinate, and *z*-coordinate directions. For convenience, denote the *x*-coordinate, *y*-coordinate, and *z*-coordinate (if in $R^{3}$) of the point ***p***_*i*_ to be interpolated as $p_{i}^{x}$, $p_{i}^{y}$, and $p_{i}^{z}$ (if in $R^{3}$), respectively. Similarly, denote the *x*-coordinate, *y*-coordinate, and *z*-coordinate (if in $R^{3}$) of the point $p_{i}^{\left(k\right)}$ in the *k*-th iteration as $p_{i}^{\left(k,x\right)}$, $p_{i}^{\left(k,y\right)}$, and $p_{i}^{\left(k,z\right)}$. Denote the *x*-coordinate, *y*-coordinate, and *z*-coordinate (if in $R^{3}$) of the control point adjustment vector $\delta_{i}^{\left(k\right)}$ as $\delta_{i}^{\left(k,x\right)}$, $\delta_{i}^{\left(k,y\right)}$, and $\delta_{i}^{\left(k,z\right)}$.

Next, we will use the interpolation curve in $R^{2}$ as an example to analyze the coordinate decomposition process of the PIA method in detail. For the case of $R^{3}$, the derivation process is similar to that of $R^{2}$.

From an algebraic perspective, the iterative process of updating the control points in the PIA method can be viewed as separate iterations in the *x*− and *y*− directions, i.e.,

$$\begin{array}{c} p_{i}^{\left(k+1,x\right)}=p_{i}^{\left(k,x\right)}+\delta_{i}^{\left(k,x\right)},\;p_{i}^{\left(k+1,y\right)}=p_{i}^{\left(k,y\right)}+\delta_{i}^{\left(k,y\right)}. \end{array}$$ (7)

**Fig 1:**
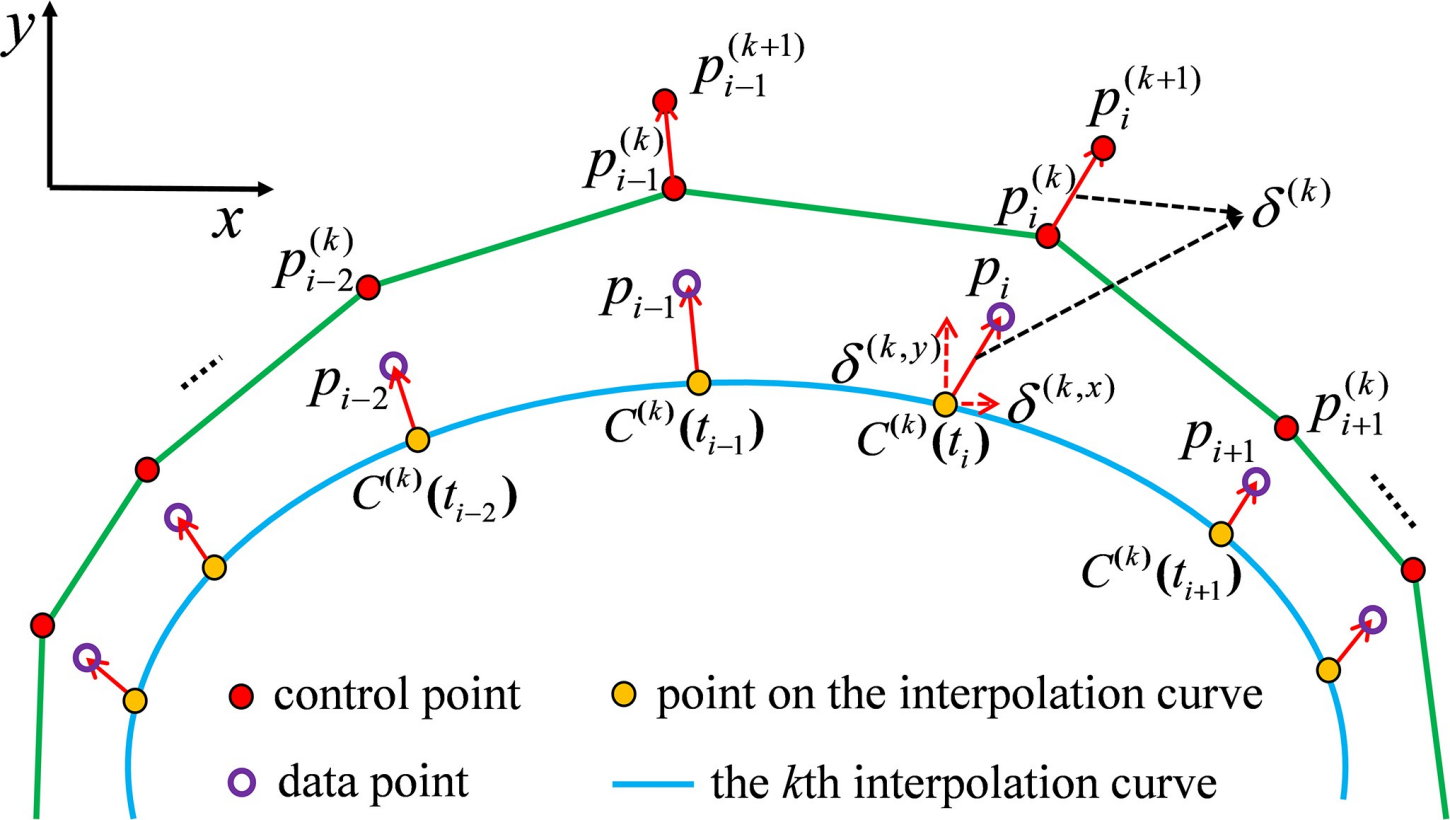
Coordinate decomposition diagram of the adjustment vector of the *i*-th control point.

From a geometric perspective, Fig 1 shows the decomposition diagram of the control point adjustment vectors along the *x*− and *y*− directions.

To achieve fast and precise adjustments of control points for the interpolation curve, we in this paper introduce an innovation upon the foundation of Eq (7). We assign unique weight coefficients to the adjustment vectors in each direction, thereby providing more flexible control. The improved iterative process can be expressed as follows:

$$\begin{array}{c} p_{i}^{\left(k+1,x\right)}=p_{i}^{\left(k,x\right)}+\omega^{\left(k,x\right)}\delta_{i}^{\left(k,x\right)},\;p_{i}^{\left(k+1,y\right)}=p_{i}^{\left(k,y\right)}+\omega^{\left(k,y\right)}\delta_{i}^{\left(k,y\right)}, \end{array}$$ (8)

where *ω*^(*k*,*x*)^ and *ω*^(*k*,*y*)^ are adjustable weight coefficients dependent on the iteration step *k*. We refer to the enhanced iterative process (8) as the weighted progressive iterative approximation based on coordinate decomposition (WPIA-CD). By introducing direction-specific weight coefficients, this method enables more precise and adaptable control of the interpolating curve.

**Remark 1** *It is straightforward to verify that the WPIA-CD method simplifies to the classical PIA method when ω*^(*k*,*x*)^ = *ω*^(*k*,*y*)^ = 1. *Furthermore, when* $\omega^{\left(k,x\right)}=\omega^{\left(k,y\right)}=\frac{2}{1+\lambda_{n}}$, *the WPIA-CD method reduces to the WPIA method*.

### 3.2 Selection of adaptive weight coefficients

In this subsection, we provide an in-depth analysis of the methodology for selecting the weight coefficients *ω*^(*k*,*x*)^ and *ω*^(*k*,*y*)^. For the sake of brevity, this paper presents a detailed exposition of the derivation process for the selection of *ω*^(*k*,*x*)^. Through this process, a more comprehensive understanding of how to reasonably set the weight coefficients to optimize the adjustment effect of the interpolating curve can be achieved.

Consider the matrix form of the iterative process in the *x*-direction

$$\begin{array}{c} P^{\left(k+1,x\right)}=P^{\left(k,x\right)}+\omega^{\left(k,x\right)}\Delta^{\left(k,x\right)}, \end{array}$$ (9)

where

$$\begin{array}{c} \Delta^{\left(k,x\right)}=P^{\left(x\right)}-BP^{\left(k,x\right)} \end{array}$$ (10)

is the component of the control point adjustment vector **Δ**^(*k*)^ along the *x*-coordinate, which is also the component of the difference vector between the current interpolating curve and the data points along the *x*-coordinate. From (9) and (10), the component of the difference vector in the next iteration along the *x*-coordinate can be expressed as

$$\begin{array}{c} \begin{array}{cc} \Delta^{\left(k+1,x\right)} & =P^{\left(x\right)}-BP^{\left(k+1,x\right)}\\ & =P^{\left(x\right)}-B\left(P^{\left(k,x\right)}+\omega^{\left(k,x\right)}\Delta^{\left(k,x\right)}\right)\\ & =\left(I-\omega^{\left(k,x\right)}B\right)\Delta^{\left(k,x\right)} \end{array} \end{array}$$

The weight coefficient *ω*^(*k*,*x*)^ in (9) can be determined by minimizing the square of the *l*_2_ norm of **Δ**^(*k*+1,*x*)^, that is

$$\begin{array}{c} \omega^{\left(k,x\right)}=\;\text{arg}\;\underset{\omega^{\left(k,x\right)}\in R}{\text{min}}\;{\left\|\Delta^{\left(k+1,x\right)}\right\|}_{2}^{2}. \end{array}$$

Let $f\left(\omega \right)=\|\left(I-\omega B\right)\Delta^{\left(k,x\right)}{\|}_{2}^{2}$, then we have

$$\begin{array}{c} \begin{array}{cc} f(\omega ) & ={\left[\left(I-\omega B\right)\Delta^{\left(k,x\right)}\right]}^{T}\left[\left(I-\omega B\right)\Delta^{\left(k,x\right)}\right]\\ & =\|\Delta^{\left(k,x\right)}{\|}_{2}^{2}-2\omega {\left(B\Delta^{\left(k,x\right)}\right)}^{T}\Delta^{\left(k,x\right)}+\omega^{2}{\left\|B\Delta^{\left(k,x\right)}\right\|}_{2}^{2} \end{array} \end{array}$$

Setting the gradient of *f*(*ω*) to zero, we can obtain the optimal weight coefficient for updating the control points in the *x*−direction.

$$\begin{array}{c} \omega^{\left(k,x\right)}=\frac{{\left(B\Delta^{\left(k,x\right)}\right)}^{T}\Delta^{\left(k,x\right)}}{\|B\Delta^{\left(k,x\right)}{\|}_{2}^{2}}. \end{array}$$ (11)

Similarly, the optimal weight coefficient for updating the control points in the *y*−direction, *ω*^(*k*,*y*)^, can be expressed as

$$\begin{array}{c} \omega^{\left(k,y\right)}=\frac{{\left(B\Delta^{\left(k,y\right)}\right)}^{T}\Delta^{\left(k,y\right)}}{\|B\Delta^{\left(k,y\right)}{\|}_{2}^{2}}. \end{array}$$ (12)

From (11) and (12), it is evident that the optimal weight coefficients for the (*k* + 1)-th iteration of control points depend on the norms of the adjustment vectors of each component in the *k*-th iteration. Dynamically adjusting the weight coefficients based on the convergence status during the iterative process allows the algorithm to better adapt to different iterative stages and data characteristics, thereby further improving the convergence speed.

**Remark 2** *In the existing weighted methods, to ensure the fastest convergence speed of the iterative method, it is usually necessary to compute the eigenvalues of the collocation matrix, as mentioned in* [3, 11]. *Although there are practical methods to solve for the weight coefficients, these methods inevitably affect the convergence speed of the iterative method. In* [15], *the authors employ optimization algorithms to find approximately optimal weight coefficients. Additionally, some studies, such as* [4], *do not provide specific theoretical optimal weight coefficients. However, for the method mentioned above, it is only necessary to calculate the norms of the difference vectors, which undoubtedly significantly reduces the computational load*.

To ensure the efficiency of the iterative algorithm, a strategy is implemented in the iterations of the control points in the *x*− and *y*− directions: during the iterative process, if the iteration error in any direction meets the termination condition (i.e., $\|\Delta^{\left(k,x\right)}{\|}_{2}^{2}<\text{tol}$ or $\|\Delta^{\left(k,y\right)}{\|}_{2}^{2}<\text{tol}$, where tol is a pre-established threshold), the iteration in that direction will immediately stop. Under this strategy, when the weight coefficients are determined according to Eqs (11) and (12) respectively, the iterative process (8) is named the adaptive progressive iterative approximation with coordinate decomposition (APIA-CD) method. The APIA-CD method for curve interpolation in a 2D space can be summarized in the following algorithm.

**Algorithm 1** Adaptive progressive iterative approximation with coordinate decomposition(APIA-CD)

1: **Input:** Set of interpolation points: ${\left\{p_{i}\right\}}_{i=0}^{n}$, pre-established threshold tol, maximum number of iterations IT_max_.

2: Set $p_{i}^{\left(0\right)}=p_{i},i=0,\cdots ,n$ and generate the initial interpolation curve.

3: Set *k* = 1, calculate $\|\Delta^{\left(k,x\right)}{\|}_{2}^{2}$ and $\|\Delta^{\left(k,y\right)}{\|}_{2}^{2}$.

4: **while** *k* < IT_max_ **do**

5:  If $\|\Delta^{\left(k,x\right)}{\|}_{2}^{2}<\text{tol}$, stop the iteration of the *x*-coordinates of the control points.

6:  If $\|\Delta^{\left(k,y\right)}{\|}_{2}^{2}<\text{tol}$, stop the iteration of the *y*-coordinates of the control points.

7:  Calculate *ω*^(*k*,*x*)^ and *ω*^(*k*,*y*)^ according to Eqs (11) and (12), respectively.

8:  Update the x- and y-coordinates of the control points according to Eq (8).

9:  Calculate $\|\Delta^{\left(k,x\right)}{\|}_{2}^{2}$ and $\|\Delta^{\left(k,y\right)}{\|}_{2}^{2}$.

10:  *k* = *k* + 1.

11: **end while**

12: **Output:** The control points $p_{i}^{\left(k\right)}$ at iteration *k*, number of iterations, and interpolation error.

#### 3.2.1 Convergence analysis

**Theorem 1** *The APIA-CD method for curves corresponding to normalized totally positive basis functions is convergent*.

**Proof** *Consider the iterative process defined by the APIA-CD method. Specifically, the weight coefficients in the x-direction is chosen to minimize the squared l*_2_ *norm of* **Δ**^(*k*+1,*x*)^, *which is a measure of the error in the x-direction, i.e*.,

$$\begin{array}{c} \omega^{\left(k,x\right)}=\;\text{arg}\;\underset{\omega^{\left(k,x\right)}\in R}{\text{min}}\;{\left\|\Delta^{\left(k+1,x\right)}\right\|}_{2}^{2}, \end{array}$$

*where* **Δ**^(*k*+1,*x*)^ *represents the x-component of the difference vector between the current interpolating curve and the data points after k* + 1 *iterations*.

*According to Remark 1, the choice of ω*^(*k*,*x*)^ *ensures that the l*_2_ *norm of* **Δ**^(*k*+1,*x*)^ *obtained by the APIA-CD method is less than or equal to the l*_2_-*norm of* **Δ**^(*k*+1,*x*)^ *obtained by the WPIA method, as described in Lemma 1. This implies that the APIA-CD method reduces the error in the x-direction at least as effectively as the WPIA method*.

*Furthermore, as established in* [1], *the WPIA method is convergent for curves corresponding to normalized totally positive basis functions. Since the APIA-CD method reduces the error at least as effectively as the WPIA method in each iteration, it follows that the APIA-CD method must also converge for curves corresponding to normalized totally positive basis functions*.

### 3.3 The surface case

Given a set of data points ${\left\{p_{ij}\right\}}_{i=0,1,\cdots ,n}^{j=0,1,\cdots ,m}\subseteq R^{3}$ to be interpolated, each point ***p***_*ij*_ is associated with a pair of parameters (*u*_*i*_, *v*_*j*_). Using these points as control points, an initial interpolation surface can be defined as

$$S^{\left(0\right)}\left(u,v\right)=\sum_{i=0}^{m}\sum_{j=0}^{n}p_{ij}^{\left(0\right)}b_{i}^{m}\left(u\right)b_{j}^{n}\left(v\right),$$

where ${\left\{b_{i}^{m}\left(u\right)\right\}}_{i=0}^{m}$ and ${\left\{b_{j}^{n}\left(v\right)\right\}}_{j=0}^{n}$ denote two sets of basis functions in the *u* and *v* parametric directions, respectively.

Let ***S***^(*k*)^(*u*, *v*) denote the interpolation surface that approximates the data points ${\left\{p_{ij}\right\}}_{i=0,1,\cdots ,n}^{j=0,1,\cdots ,m}$ after *k* iterations (*k* = 0, 1, ⋯). The adjustment vector at the (*i*, *j*)-th data point for the (*k* + 1)-th iteration is given by

$$\delta_{ij}^{\left(k+1\right)}=p_{ij}-S^{\left(k\right)}\left(u_{i},v_{j}\right).$$

By decomposing $\delta_{ij}^{\left(k+1\right)}$ along the coordinate axes, we obtain the components $\delta_{ij}^{\left(k+1,x\right)}$, $\delta_{ij}^{\left(k+1,y\right)}$ and $\delta_{ij}^{\left(k+1,z\right)}$, respectively. Consequently, the coordinates of the control points $p_{ij}^{\left(k+1\right)}$ for the subsequent iteration of the interpolation surface are updated as

$$\begin{array}{c} p_{ij}^{\left(k+1,x\right)}=p_{ij}^{\left(k,x\right)}+\omega^{\left(k,x\right)}\delta_{ij}^{\left(k+1,x\right)}, \end{array}$$

$$\begin{array}{c} p_{ij}^{\left(k+1,y\right)}=p_{ij}^{\left(k,y\right)}+\omega^{\left(k,y\right)}\delta_{ij}^{\left(k+1,y\right)}, \end{array}$$

and

$$\begin{array}{c} p_{ij}^{\left(k+1,z\right)}=p_{ij}^{\left(k,z\right)}+\omega^{\left(k,z\right)}\delta_{ij}^{\left(k+1,z\right)}. \end{array}$$

In this way, a sequence of surfaces ***S***^(*k*)^(*u*, *v*) that successively approximate the data points ${\left\{p_{ij}\right\}}_{i=0,1,\cdots ,n}^{j=0,1,\cdots ,m}$ can be constructed for *k* = 0, 1, ⋯. This iterative methodology for generating sequences of interpolation surfaces is referred to as the weighted progressive iterative approximation based on coordinate decomposition (WPIA-CD) for surface cases.

Next, we discuss the selection of the weights *ω*^(*k*,*x*)^, *ω*^(*k*,*y*)^ and *ω*^(*k*,*z*)^. Again, only the derivation of the choice of *ω*^(*k*,*x*)^ is described.

First, we organize the interpolation points, control points, and the components of the adjustment vectors along the *x*-coordinate into vector forms

$$P^{\left(x\right)}={\left[p_{00}^{\left(x\right)},p_{01}^{\left(x\right)},\cdots ,p_{0n}^{\left(x\right)},p_{10}^{\left(x\right)},p_{11}^{\left(x\right)},\cdots ,p_{1n}^{\left(x\right)},\cdots ,p_{m0}^{\left(x\right)},p_{m1}^{\left(x\right)},\cdots ,p_{mn}^{\left(x\right)}\right]}^{T},$$

$$P^{\left(k,x\right)}={\left[p_{00}^{\left(k,x\right)},p_{01}^{\left(k,x\right)},\cdots ,p_{0n}^{\left(k,x\right)},p_{10}^{\left(k,x\right)},p_{11}^{\left(k,x\right)},\cdots ,p_{1n}^{\left(k,x\right)},\cdots ,p_{m0}^{\left(k,x\right)},p_{m1}^{\left(k,x\right)},\cdots ,p_{mn}^{\left(k,x\right)}\right]}^{T},$$

and

$$\Delta^{\left(k,x\right)}={\left[\delta_{00}^{\left(k,x\right)},\delta_{01}^{\left(k,x\right)},\cdots ,\delta_{0n}^{\left(k,x\right)},\delta_{10}^{\left(k,x\right)},\delta_{11}^{\left(k,x\right)},\cdots ,\delta_{1n}^{\left(k,x\right)},\cdots ,\delta_{m0}^{\left(k,x\right)},\delta_{m1}^{\left(k,x\right)},\cdots ,\delta_{mn}^{\left(k,x\right)}\right]}^{T}.$$

Note that **Δ**^(*k*,*x*)^ can be equivalently expressed as

$$\Delta^{\left(k,x\right)}=Q^{\left(x\right)}-\left(B_{1}⊗B_{2}\right)P^{\left(k,x\right)},$$

where ⊗ denotes the Kronecker product of matrices, $B_{1}={\left[b_{i}\left(u_{j}\right)\right]}_{i,j=0}^{m,m}$ and $B_{2}={\left[b_{i}\left(u_{j}\right)\right]}_{i,j=0}^{n,n}$ are the collocation matrices of the basis functions ${\left\{b_{i}\left(u\right)\right\}}_{i=0}^{m}$ and ${\left\{b_{i}\left(v\right)\right\}}_{i=0}^{n}$ evaluated at parameters *u*_*j*_ for *j* = 0, 1, ⋯, *m* and *v*_*j*_ for *j* = 0, 1, ⋯, *n*, respectively.

The iterative process of updating of control points along the *x*-direction can be expressed as a matrix form

$$\begin{array}{c} P^{\left(k+1,x\right)}=P^{\left(x\right)}+\left[I-\left(B_{1}⊗B_{2}\right)\right]P^{\left(k,x\right)}=P^{\left(k,x\right)}+\Delta^{\left(k,x\right)}, \end{array}$$ (13)

where *I* is a identity matrix of the same order as *B*_1_ ⊗ *B*_2_.

The adjustment vector of the (*k* + 1)-th iteration along the *x*-direction can be expressed as

$$\begin{array}{c} \begin{array}{cc} \Delta^{\left(k+1,x\right)} & =Q^{\left(x\right)}-\left(B_{1}⊗B_{2}\right)P^{\left(k+1,x\right)}\\ & =P^{\left(x\right)}-\left(B_{1}⊗B_{2}\right)\left(P^{\left(k,x\right)}+\omega^{\left(k,x\right)}\Delta^{\left(k,x\right)}\right)\\ & =\left[I-\omega^{\left(k,x\right)}\left(B_{1}⊗B_{2}\right)\right]\Delta^{\left(k,x\right)} \end{array} \end{array}$$ (14)

In analogy with the curve interpolation, the weight *ω*^(*k*,*x*)^ in (14) is ascertained by minimizing the square of the *l*_2_-norm of **Δ**^(*k*+1,*x*)^, i.e.,

$$\begin{array}{c} \omega^{\left(k,x\right)}=\;\text{arg}\;\underset{\omega^{\left(k,x\right)}\in R}{\text{min}}\;{\left\|\Delta^{\left(k+1,x\right)}\right\|}_{2}^{2}. \end{array}$$ (15)

Letting $g\left(\omega \right)=\|\left[I-\omega \left(B_{1}⊗B_{2}\right)\right]\Delta^{\left(k,x\right)}{\|}_{2}^{2}$, we have

$$\begin{array}{c} \begin{array}{cc} g(\omega ) & ={\left[\left[I-\omega \left(B_{1}⊗B_{2}\right)\right]\Delta^{\left(k,x\right)}\right]}^{T}\left[\left[I-\omega \left(B_{1}⊗B_{2}\right)\right]\Delta^{\left(k,x\right)}\right]\\ & =\|\Delta^{\left(k,x\right)}{\|}_{2}^{2}-2\omega {\left[\left(B_{1}⊗B_{2}\right)\Delta^{\left(k,x\right)}\right]}^{T}\Delta^{\left(k,x\right)}+\omega^{2}{\left\|\left(B_{1}⊗B_{2}\right)\Delta^{\left(k,x\right)}\right\|}_{2}^{2} \end{array} \end{array}$$ (16)

Setting the gradient of *g*(*ω*) to 0, the optimal weight *ω*^(*k*,*x*)^ for updating the control points in the *x*-direction can be given by

$$\begin{array}{c} \omega^{\left(k,x\right)}=\frac{{\left(\left(B_{1}⊗B_{2}\right)\Delta^{\left(k,x\right)}\right)}^{T}\Delta^{\left(k,x\right)}}{\|\left(B_{1}⊗B_{2}\right)\Delta^{\left(k,x\right)}{\|}_{2}^{2}}. \end{array}$$ (17)

Similarly, the optimal weight coefficients for updating the control points in the *y*- and *z*- directions can be respectively expressed as

$$\begin{array}{c} \omega^{\left(k,y\right)}=\frac{{\left(\left(B_{1}⊗B_{2}\right)\Delta^{\left(k,y\right)}\right)}^{T}\Delta^{\left(k,y\right)}}{\|\left(B_{1}⊗B_{2}\right)\Delta^{\left(k,y\right)}{\|}_{2}^{2}} \end{array}$$

and

$$\begin{array}{c} \omega^{\left(k,z\right)}=\frac{{\left(\left(B_{1}⊗B_{2}\right)\Delta^{\left(k,z\right)}\right)}^{T}\Delta^{\left(k,z\right)}}{\|\left(B_{1}⊗B_{2}\right)\Delta^{\left(k,z\right)}{\|}_{2}^{2}}. \end{array}$$

To guarantee the computational efficiency of the surface interpolation algorithm, the iterative processes along the *x*-, *y*-, and *z*- directions of updating the control points are stopped as soon as the error associated with the iteration in any given direction falls below a pre-established threshold. The optimal weights are dynamically adjusted depending on the norms of adjustment vectors at the current iteration. This adaptive approach is referred to as the adaptive progressive iterative approximation based on coordinate decomposition (AWPIA-CD) for surface interpolation.

## 4 Numerical results

In this section, a series of numerical examples are presented to assess the efficiency of APIA-CD in dealing with geometric approximation problems. In order to comprehensively evaluate the performance of APIA-CD in different application scenarios, the experiments cover both uniformly and non-uniformly sampled data sets in $R^{2}$ and $R^{3}$, including the application of APIA-CD to Bézier curves (surfaces) and quasi-uniform cubic B-spline curves (surfaces).

To evaluate the accuracy of the approximate interpolation algorithms for curve and surface cases, we respectively use

$$\epsilon_{\text{cur}}^{\left(k\right)}=\underset{1\leq i\leq n}{\text{max}}\;\left\|\delta_{i}^{\left(k\right)}\right\|\;\text{and}\;\epsilon_{\text{surf}}^{\left(k\right)}=\underset{\begin{array}{c} 1\leq i\leq m\\ 1\leq j\leq n \end{array}}{\text{max}}\;\left\|\delta_{ij}^{\left(k\right)}\right\|$$

to measure the interpolation error at the *k*-the iteration.

For comparison, we also tested some other variants of PIA with weights, including the WPIA method [3] and the DWPIA method [4]. All numerical experiments were performed on a computer with an Intel(R) Core(TM) i7–8565U CPU @ 1.80GHz 1.99 GHz processor and 8.00 GB of RAM.

### 4.1 Numerical results of curve interpolation

**Example 1** 30 *points are sampled from the function* $y=e^{-{\left(x-3\right)}^{2}}\;\text{sin}\;x$ *where* 0 ≤ *x* ≤ 2*π*.

**Example 2** 49 *data points are sampled uniformly from the four leaf clover function*:

$$\left\{ \begin{array}{c} x=4\;\text{sin}(t)\;\text{sin}(4t),\\ y=4\;\text{sin}(4t)\;\text{cos}(t),\; \end{array}-\pi \leq t\leq \pi .\right.$$

**Example 3** 53 *data points from the parametric equations*

$$\left\{ \begin{array}{c} x=\text{sin}(3t)\;\text{cos}(t),\\ y=\text{sin}(3t)\;\text{sin}(t),\\ z=t, \end{array}-\pi \leq t\leq \pi .\right.$$

**Fig 2:**
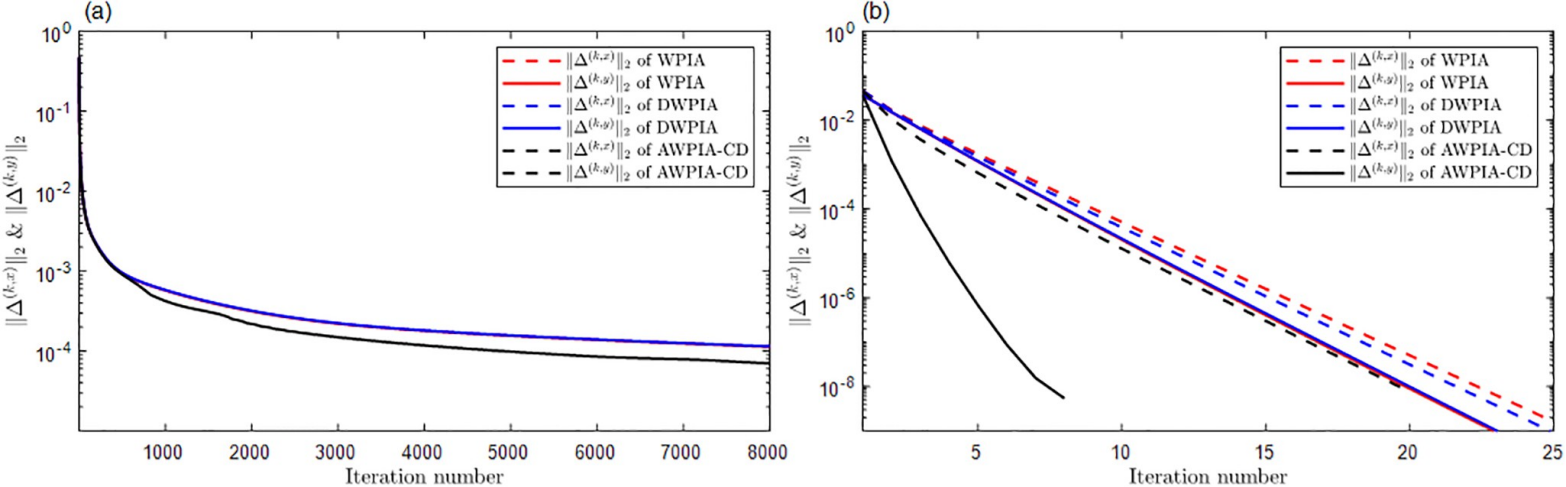
The relationship between the norms of each component of the adjusted vector and the number of iterations in Example 1. (a) Bézier surface. (b) Cubic B-spline surface.

**Fig 3:**
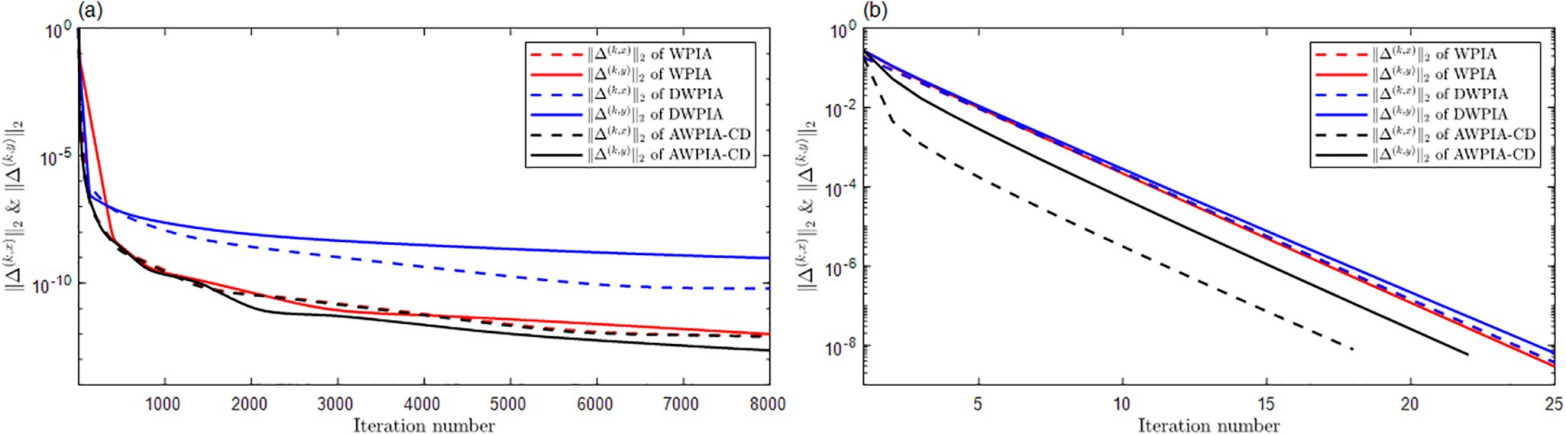
The relationship between the norms of each component of the adjusted vector and the number of iterations in Example 2. (a) Bézier surface. (b) Cubic B-spline surface.

**Fig 4:**
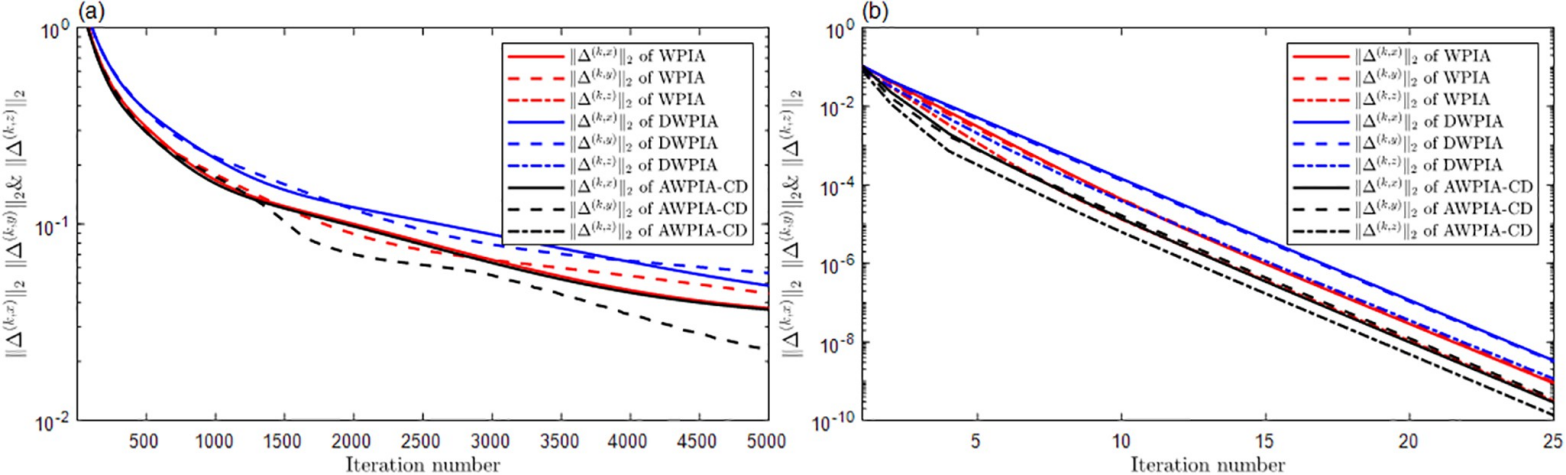
The relationship between the norms of each component of the adjusted vector and the number of iterations in Example 3. (a) Bézier surface. (b) Cubic B-spline surface.

For curve interpolation, we employed WPIA, DWPIA, and APIA-CD to iteratively interpolate the given data points in Examples 1–3. In our experiments, we individually assessed the convergence behavior for different coordinate components. To visually demonstrate the performance of the algorithms, Figs 2 to 4 show the *l*_2_-norm of each component of the difference vectors as a function of the number of iterations. The results showed that the norm of each component of the difference vector exhibited varying convergence behaviors. This variation in convergence rates is expected and can be attributed to several factors. Firstly, different coordinate components may correspond to different directions along the curve, and the distribution of control points in these directions may be uneven, leading to differential sensitivity to adjustments. Secondly, the interpolation algorithm may be more sensitive to certain components, necessitating more frequent updates for these parts. Furthermore, the strategy for selecting weights can significantly influence the convergence rates of the components. For instance, components with higher weights will undergo more significant adjustments during the iterations. The variation in convergence behavior is crucial for the design and optimization of the iterative method because it allows the algorithm to adjust weights flexibly according to the actual convergence of each component, thereby more effectively controlling the approximation process and ensuring that the final result meets accuracy requirements and converges within a reasonable number of iterations.

The proposed algorithm takes into account both the overall approximation effect and the convergence characteristics of each component. By flexibly adjustment, the performance of the approximation algorithm can be optimized to achieve more efficient and accurate geometric modeling. Moreover, whether for Bézier curves or B-spline curves, the APIA-CD method has shown faster rapid convergence, which is due to the dynamic adjustment of weights during the iterative process of APIA-CD, thereby more effectively controlling the approximation process.

**Fig 5:**
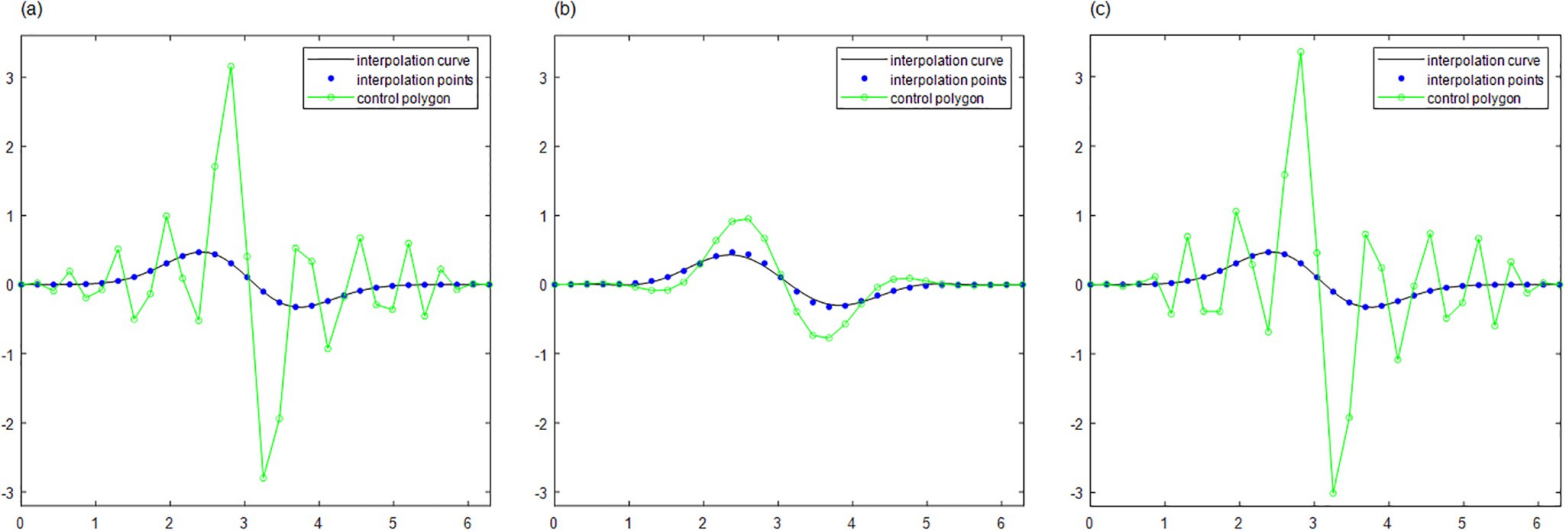
Bézier curves by AWPIA-CD with different iterations when interpolating the points in Example 1. (a) $k=1(\epsilon_{\text{cur}}^{\left(k\right)}=7.06\times 10^{-2})$. (b) $k=2(\epsilon_{\text{cur}}^{\left(k\right)}=5.83\times 10^{-2})$. (c) $k=5000(\epsilon_{\text{cur}}^{\left(k\right)}=3.76\times 10^{-5})$.

**Fig 6:**
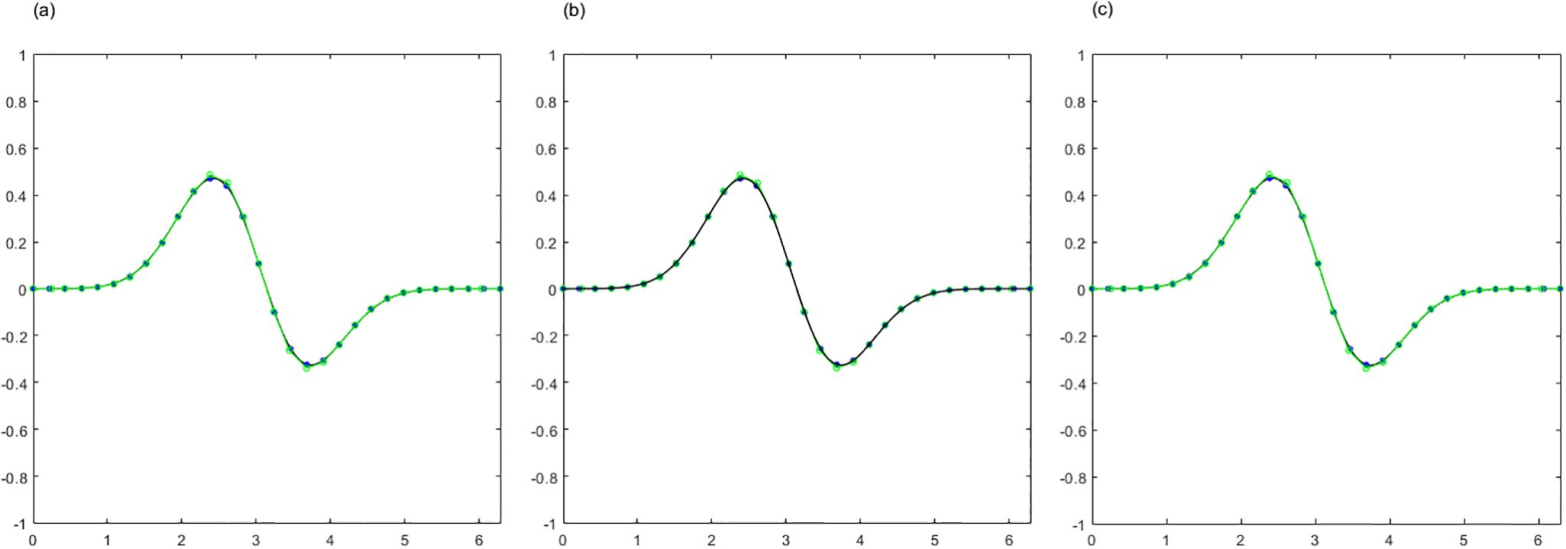
Cubic B-spline curves by AWPIA-CD with different iterations when interpolating the points in Example 1. (a) $k=1(\epsilon_{\text{cur}}^{\left(k\right)}=4.53\times 10^{-3})$. (b) $k=2(\epsilon_{\text{cur}}^{\left(k\right)}=1.23\times 10^{-3})$. (c) $k=35(\epsilon_{\text{cur}}^{\left(k\right)}=3.17\times 10^{-14})$.

**Fig 7:**
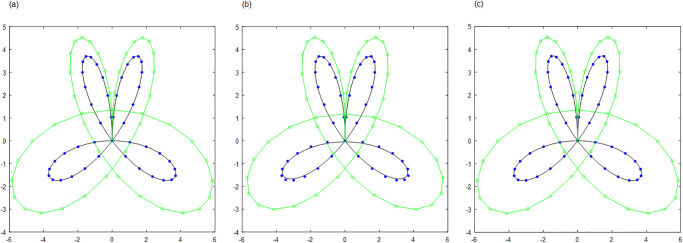
Bézier curves by AWPIA-CD with different iterations when interpolating the points in Example 2. (a) $k=1(\epsilon_{\text{cur}}^{\left(k\right)}=2.60\times 10^{-1})$. (b) $k=2(\epsilon_{\text{cur}}^{\left(k\right)}=7.99\times 10^{-2})$. (c) $k=5000(\epsilon_{\text{cur}}^{\left(k\right)}=5.13\times 10^{-13})$.

**Fig 8:**
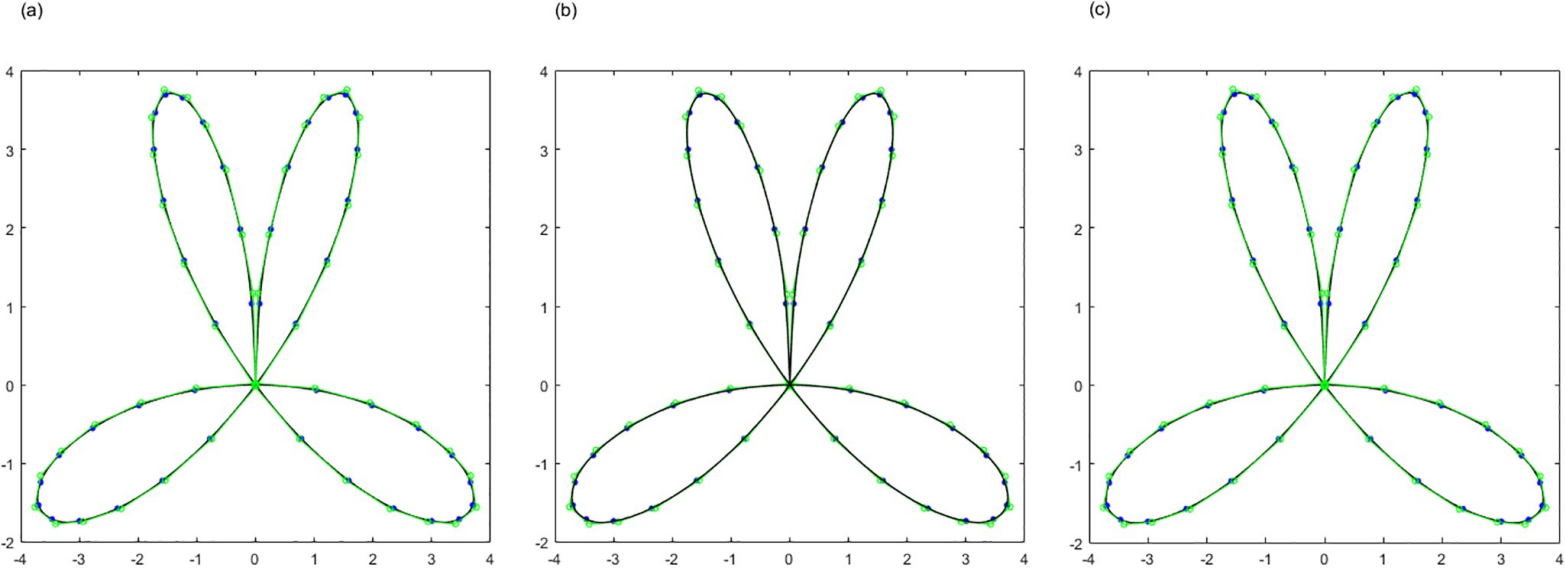
Cubic B-spline curves by AWPIA-CD with different iterations when interpolating the points in Example 2. (a) $k=1(\epsilon_{\text{cur}}^{\left(k\right)}=2.91\times 10^{-2})$. (b) $k=2(\epsilon_{\text{cur}}^{\left(k\right)}=6.86\times 10^{-3})$. (c) $k=40(\epsilon_{\text{cur}}^{\left(k\right)}=2.42\times 10^{-14})$.

**Fig 9:**
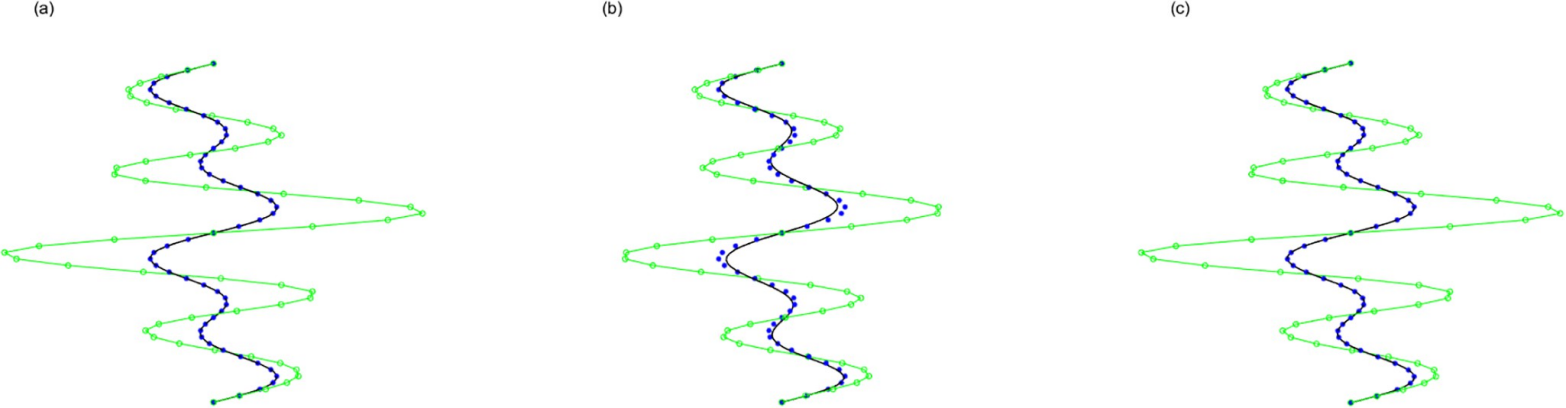
Bézier curves by AWPIA-CD with different iterations when interpolating the points in Example 3. (a) $k=1(\epsilon_{\text{cur}}^{\left(k\right)}=2.79\times 10^{-1})$. (b) $k=2(\epsilon_{\text{cur}}^{\left(k\right)}=1.24\times 10^{-1})$. (c) $k=5000(\epsilon_{\text{cur}}^{\left(k\right)}=5.06\times 10^{-9})$.

**Fig 10:**
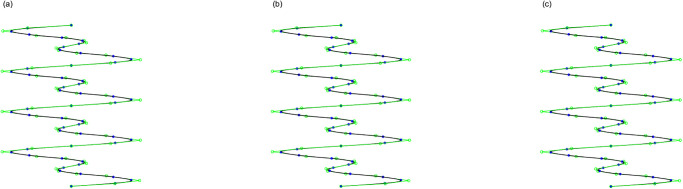
Cubic B-spline curves by AWPIA-CD with different iterations when interpolating the points in Example 3. (a) $k=1(\epsilon_{\text{cur}}^{\left(k\right)}=2.91\times 10^{-2})$. (b) $k=2(\epsilon_{\text{cur}}^{\left(k\right)}=8.56\times 10^{-3})$. (c) $k=34(\epsilon_{\text{cur}}^{\left(k\right)}=2.55\times 10^{-14})$.

By employing the APIA-CD method, the point sets presented in Examples 1 to 3 are approximately interpolated using Bézier curves and cubic B-spline curves. These point sets and their corresponding interpolation curves are illustrated in Figs 5 to 10, showcasing the progression of curves after various iterations. Within Figs 5 to 10, the blue points denote the interpolation points, the black curves represent the interpolated curves, and the green lines indicate the control polygons. To maintain clarity and simplicity in the figures, a legend is included only in Fig 5, with the understanding that the same color-coding applies to the subsequent figures. To complement these visual representations, the interpolation errors are detailed below their respective figures. It is evident that the curves derived from APIA-CD progressively approximate the provided data points with increasing accuracy.

### 4.2 Numerical results of surface interpolation

**Example 4** *Consider interpolating* 20 *points*: (1, 1, 1), (1, 2, 4), (1, 3, 2), (1, 4, 2), (1, 5, 2), (2, 1, 2), (2, 2, 2), (2, 3, 3), (2, 4, 6), (2, 5, 4), (3, 1, 3), (3, 2, 2), (3, 3, 4), (3, 4, 4), (3, 5, 3), (4, 1, 4), (4, 2, 6), (4, 3, 1), (4, 4, 1), (4, 5, 2).

**Example 5** *Consider interpolating* 40 × 42 *points sampled from the peaks function*.

**Fig 11:**
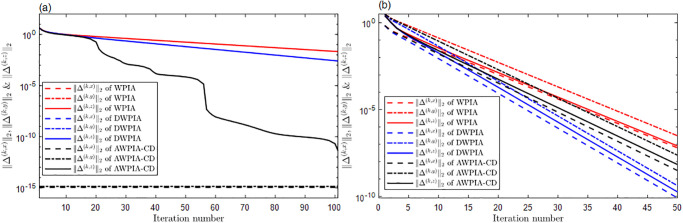
The relationship between the norms of each component of the adjusted vector and the number of iterations in Example 4. (a) Bézier surface. (b) Bi-cubic B-spline surface.

**Fig 12:**
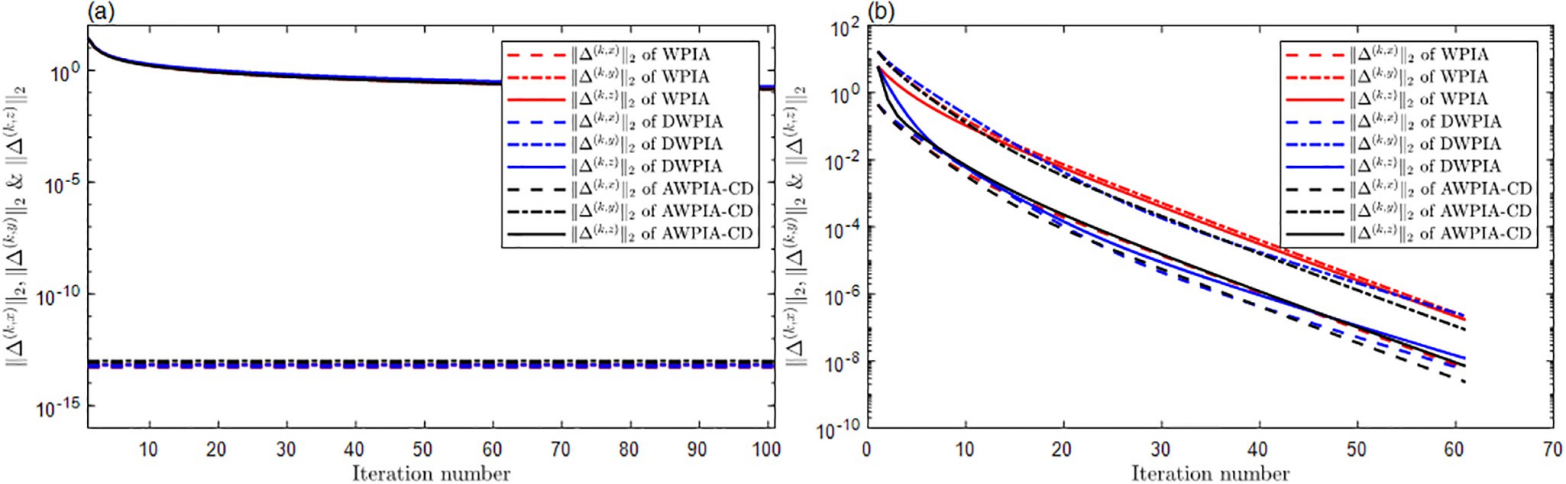
The relationship between the norms of each component of the adjusted vector and the number of iterations in Example 5. (a) Bézier surface. (b) Bi-cubic B-spline surface.

For surface interpolation, we we have also employed WPIA, DWPIA, and AWPIA-CD to iteratively interpolate the given data points in Examples 4 and 5. Similar to the curve case, we individually assessed the convergence behavior of different coordinate components in our experiments and visually demonstrated the performance of the algorithms in Figs 11 and 12. We observed that the convergence behaviors of the norms of different coordinate components also vary in surface interpolation. Specifically, the norms of the components of the difference vector along the *x*− and *y*− directions rapidly approach machine precision after the first iteration, indicating a swift convergence of the algorithm. During the iterative process, it is only necessary to adjust the *z*-coordinate of the control points, which will obviously reduce the computational load. Moreover, we listed in Tables 1 and 2 the number of iterations, the interpolation errors, and the runtime of WPIA, DWPIA, and AWPIA-CD. From Tables 1 and 2, we can see that under the requirement of the same accuracy, AWPIA-CD requires fewer iterations than WPIA and DWPIA, and the runtime of AWPIA-CD are less than those of WPIA and DWPIA. These results highlight the efficiency and effectiveness of AWPIA-CD in terms of iteration count and runtime.

**Table 1:** Interpolation errors and runtime of AWPIA-CD and other methods for Bézier surface in Example 5.

	WPIA		DWPIA		AWPIA-CD	
*k*	*ε* ^(*k*)^	*T*(s)	*ε* ^(*k*)^	*T*(s)	*ε* ^(*k*)^	*T*(s)
1	1.13e+00	–	1.14e+00	–	1.19e+00	–
2	6.87e-01	–	6.86e-01	–	6.79e-01	–
5	3.82e-01	–	3.82e-01	–	3.82e-01	–
10	2.36e-01	–	2.35e-01	–	2.31e-01	–
20	1.26e-01	–	1.26e-01	–	1.24e-01	–
50	4.76e-02	–	4.76e-02	–	4.73e-02	–
100	2.19e-02	–	2.19e-02	–	2.12e-02	–
1000	1.52e-03	–	1.52e-03	–	7.76e-04	2.98e-01
1804	7.74e-04	8.85e-01	–	–	–	–
1808	–	–	7.76e-04	4.62e+00	–	–

**Table 2:** Interpolation errors and runtime of AWPIA-CD and other methods for bi-cubic B-spline surface in Example 5.

	WPIA		DWPIA		AWPIA-CD	
*k*	*ε* ^(*k*)^	*T*(s)	*ε* ^(*k*)^	*T*(s)	*ε* ^(*k*)^	*T*(s)
1	1.30e-01	–	7.86e-02	–	4.81e-03	–
2	9.43e-02	–	3.45e-02	–	2.19e-03	–
5	3.65e-02	–	3.02e-03	–	1.06e-04	–
10	8.11e-03	–	5.94e-05	–	1.90e-06	–
20	4.78e-04	–	1.09e-06	–	9.80e-10	–
50	2.02e-07	–	7.56e-10	–	1.97e-14	1.78e-02
101	–	–	1.76e-14	2.26e-01	–	–
118	1.69e-14	6.13e-01	–	–	–	–

**Fig 13:**
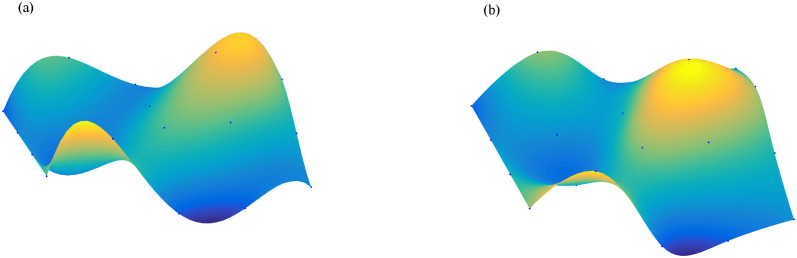
Bézier surface and bi-cubic B-spline surface obtained by AWPIA-CD for Example 4. (a) Bézier surface $\left(\epsilon_{\text{surf}}^{\left(100\right)}=2.37\times 10^{-12}\right)$. (b) Bi-cubic B-spline surface $\left(\epsilon_{\text{surf}}^{\left(81\right)}=4.13\times 10^{-14}\right)$.

**Fig 14:**
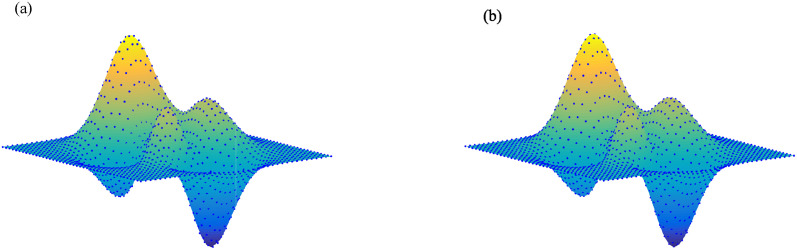
Bézier surface and bi-cubic B-spline surface obtained by AWPIA-CD for Example 5. (a) Bézier surface $\left(\epsilon_{\text{surf}}^{\left(100\right)}=2.12\times 10^{-2}\right)$. (b) Bi-cubic B-spline surface $\left(\epsilon_{\text{surf}}^{\left(52\right)}=1.34\times 10^{-14}\right)$.

By implementing APIA-CD, the point sets in Examples 4 and 5 are approximately interpolated by Bézier surfaces and bi-cubic B-spline surfaces. Figs 13 and 14 display the point sets and interpolation surfaces for Examples 4 and 5. It can be observed that both the Bézier and bi-cubic B-spline surfaces generated by AWPIA-CD interpolate the given point sets well.

## 5 Conclusions

This paper studies and optimizes the adjustment process of control vertices in the interpolated geometric iterative method. To improve control vertex adjustment, the strategy of coordinate decomposition is used. This involves projecting the adjustment vectors to each coordinate axis separately and obtaining the components using vector operations. This paper also introduces adjustable weight coefficients for each component. Adjusting these coefficients speeds up convergence and improves accuracy. The method is flexible and can be easily adapted to different situations. The method in this paper can be used for most all-positive basis functions. These features show how flexible the method is and how accurate it is.

Experiments show that the technique improves the efficiency of the algorithm and accurately adjusts curves or surfaces to approximate data points. This research helps develop and use the geometric iterative method more widely.

## Supporting information

S1 DataThe data for Example 5 are sampled from the peaks function.$z=3{\left(1-x\right)}^{2}e^{-\left(x^{2}\right)-{\left(y+1\right)}^{2}}-10\left(\frac{x}{5}-x^{3}-y^{5}\right)e^{-x^{2}-y^{2}}-\frac{1}{3}e^{-{\left(x+1\right)}^{2}-y^{2}},$
where $x=-3+\frac{6}{39}i,i=0,1,\cdots ,39;\;y=-3+\frac{6}{41}j,j=0,1,\cdots ,41$.(RAR)
